# Supplementary material for: Are carbon emissions trading and green financial instruments synergistic? -Comprehensive quantitative research based on content analysis
Source: PLoS One. 2024 Mar 7;19(3):e0298601. doi: 10.1371/journal.pone.0298601 (PMC10919601; doi:10.1371/journal.pone.0298601)
Supplement: S1 Data — (ZIP) [file pone.0298601.s001.zip › Data/地级市Z_F工作报告.zip!/╔╜╬≈/┤≤═1⁄4╩╨/└┤╫╘╣1⁄2╓┌║┼í╛┬φ┐╦╩2╛▌═°í┐_2013.docx]

各位代表:

现在,我代表市人民政府向大会作工作报告,请予审议,并请市政协委员和其他列席人员提出意见。

一、2012年工作回顾

过去的一年,全市认真贯彻落实党的十八大精神和省委、省政府的决策部署,深入实施转型发展、绿色崛起战略,加快“三名一强”建设步伐,经济社会发展迈上新台阶。

经济保持平稳增长。年初确定的各项主要指标全面完成。全市地区生产总值同比增长10%,规模以上工业增加值增长13.4%,财政总收入增长17.6%,公共财政预算收入增长24.2%,固定资产投资增长25.1%,社会消费品零售总额增长16%,城镇居民人均可支配收入增长13.6%,农民人均纯收入增长14.3%,居民消费价格涨幅2.3%,万元GDP综合能耗下降4.83%,污染物排放实现控制目标。

项目建设步伐加快。以项目为抓手,以园区为载体,推进传统产业提升和新兴产业发展。装备制造、新医药、煤化工等工业园区集聚示范效应增强。全市签约项目152个,项目总投资2107.87亿元。同煤集团东周窑1000万吨矿井及洗煤厂项目投产,金庄1000万吨矿井完成500万吨工作面。8座资源整合重组矿井完成技改扩建。晋投1万吨玄武岩加工、华润10万吨雪花啤酒及国药威奇达、国药中抗制药搬迁改造项目竣工投产。新增风力发电装机40万千瓦,建成光伏电站4万千瓦。

城市建设日新月异。城镇化率提高1.45个百分点,达到57.95%。投资22亿元,建设改造城市道路57条103公里,城市路网体系趋于完善。天大高速、同源高速通车,广源高速基本完工,市县一小时经济圈初步形成。大同机场新航站楼投入使用。御东新区建设步伐加快,太阳宫、博物馆、大剧院、图书馆、美术馆、体育中心六大工程主体基本完成。市政设施、园林绿化、景区景点维护管理力度加大。数字城管平台作用进一步发挥,区级政府城市管理主体功能强化。住宅小区特别是政府安居工程物业管理得到加强,居民满意度不断提高。

古城修复有序推进。北城墙修复完工,西城墙修复过半,展览馆平移工程分体成功,代王府一期主体完成。云冈石窟窟檐保护工程进展顺利。晋华宫国家矿山公园正式开园,文化科技产业基地主体完成。成功举办第十一届云冈文化旅游节和国际壁画双年展、曾竹韶雕塑艺术奖学金作品展、中国大同国际汽车文化节。文化旅游业加快发展,旅游总收入163亿元,同比增长19%。

农业发展水平提升。粮食总产9.49亿公斤,连续3年创历史新高。新增设施农业4.47万亩,总面积达到16万亩。投资55亿元完成现代农业项目363个。雨润百万头生猪屠宰项目投产,新发地农产品冷链物流市场、露露集团大同绿苑公司项目进展顺利。“一县一业”、“一村一品”稳步发展,5个县、322个村被列入省级基地县、专业村。孤山水库竣工,唐河水库蓄水,守口堡水库开工,新建农村饮水安全工程105处,105个村8.92万农村人口的饮水安全状况得到改善。移民扶贫搬迁9450人。农村公共事业新的“五个全覆盖”工程全面完成。

生态环境质量改善。新增集中供热面积600万平方米,市区二级以上天数达到349天,比上年增加2天。御东污水处理厂和东郊、西郊污水处理厂改扩建工程基本完成,餐厨垃圾处理厂开工建设。完成造林47.44万亩,全省造林绿化现场会在大同召开。6.8平方公里的文瀛湖生态公园建成开放,采凉山森林公园、十里河生态园、口泉植物园、御河东西两岸景观等重点绿化工程基本完成,建成区新增绿化面积346万平方米,绿化覆盖率、绿地率、人均公共绿地分别比上一年增加3.2个百分点、2.48个百分点、1.23平方米。

社会民生全面进步。开工建设保障性住房11.6万套,基本建成4.9万套。大同三中、大同十六中、大同实验小学、大同卫校4所新校投入使用。新改扩建20所标准化幼儿园。调整市直学区并组建5个教育集团。阳高、天镇、大同、浑源、灵丘、广灵6县落实学生营养改善计划,惠及507所中小学校11.78万名学生。市疾控中心、一医院、六医院、中医院、光荣院等新建工程投入使用,五医院新院基本建成。城镇新增就业5.44万人,下岗失业人员再就业2.86万人,城镇登记失业率2.9%。社会保障体系不断完善,社会救助逐步规范,城乡低保实现应保尽保,各类社会保险参保人数和基金收缴均超额完成任务。安全生产形势稳定,重大安全事故为零。国防教育和双拥工作深入开展,人口计生、妇女儿童、老龄事业和红十字会、慈善等公益事业进一步加强,宗教、外事、信访、统计、气象、地震、人防和档案等各项工作都取得新的成绩。

政府建设不断加强。坚持依法行政,自觉接受人大法律监督和政协民主监督,办理人大代表建议129件、政协提案288件,办复率均为100%;提请市人大常委会审议地方性法规草案2件,制定政府规章2件。行政审批制度改革走在全省前列,行政审批和行政权力服务事项办理流程进一步优化,行政处罚自由裁量权标准进一步规范,行政监察和审计监督进一步加强。

上述成绩的取得,是省委、省政府正确领导的结果,是市委总揽全局、坚强领导的结果,是市人大、市政协监督支持的结果,是全市人民共同奋斗、顽强拼搏的结果。在此,我代表市政府,向全市工人、农民、知识分子和广大干部,向各位人大代表和政协委员,向各民主党派、人民团体和其他各界人士,向驻同解放军指战员、武警官兵和全市公安民警,向所有关心支持大同发展的同志们、朋友们,表示衷心的感谢和崇高的敬意!此时,我们还要向为大同发展作出重要贡献的耿彦波市长,表示衷心的感谢!

二、形势与任务

各位代表,我市正处于转型发展攻坚期和大有可为的战略机遇期,前进的道路上还面临许多矛盾和挑战,经济社会发展不平衡、不协调、不可持续的问题还比较突出。

一是经济总量小,财政负担重。地区生产总值不达千亿,排全省第九位,人均值比全国和全省平均水平分别低10636元和5815元。工业经济不大,增加值仅占全省6.5%;县域经济不强,总量占全省比重倒数第一;非公经济不活,增加值占GDP比重低于全省平均水平25个百分点。煤炭市场需求低迷、量价齐跌,发展面临的不确定因素增多。财政总收入、公共财政预算收入仅占全省7.2%和5.3%,财政收支矛盾日益突出,政府负债风险不容小视。

二是小康实现程度低,社会建设任务重。全面小康实现程度低于全省平均水平1.7个百分点,其中,文化教育指标低3个百分点,居民人均住房使用面积指标低12.6个百分点,社会和谐指标低16.1个百分点。因土地房屋征收征用和补偿安置、工程款和农民工工资拖欠、企业破产、环境污染等引发的矛盾明显增多。

三是市域城镇化质量低,城乡建设差距大。城乡之间二元结构差距较大,城市内部二元结构较为突出,城乡居民收入比的小康实现程度低于全省43个百分点。县城人口规模均不足10万人,3个县的城镇化率排在全省后10位。城乡规划滞后,法定规划体系不健全。城市建设红利和潜在风险同时显现,古城修复利用任务艰巨。城乡环境脏乱差现象较为严重。城镇组群式发展态势尚未形成,与在全省“一核一圈三群”城镇化战略中的地位不相称。

四是生态建设差距大,优化发展环境任务重。森林覆盖率低,有的县不足10%。空气质量有待进一步改善,污水、垃圾处理有待进一步加强。水、土地等资源约束趋紧,节能减排形势依然严峻,生态建设任重道远。一些干部思想解放不够,危机意识不强,行政效率不高,与转型跨越的要求不相称。

在正视挑战和困难的同时,我们更要看到发展的机遇和前景。城镇化是扩大内需的最大潜力,是经济增长的巨大引擎,城镇化的快速推进必将拉动经济快速发展。我省转型综改试验区建设正向纵深展开,我市一批重大改革、重大事项、重大项目已列入全省实施方案和行动计划,改革红利将集中释放。国家大力实施集中连片特困地区区域发展与扶贫攻坚规划,我市6个县列入燕山-太行山连片特困地区,对争取国家支持机遇难得;我省启动实施“百企千村”产业扶贫开发工程,将为扶贫攻坚和县域经济发展注入新的活力。随着国家京津冀鲁火电项目限批政策的出台,东部地区产业转移步伐加快,为我市电力、煤炭产业提供发展空间,我市资源、区位、文化优势将进一步凸现。积极争取开放航空口岸,将为保税区建设创造条件,为大同走向世界打开窗口。积极争取立项的大张客运专线,将促进我市全方位融入京津冀经济圈,建成北京后花园,扩大大同开放度。特别是,全市上下人心思进、人心思干,蕴藏着争先进位、负重赶超的无穷创造力和极大正能量。大同加快发展天时、地利、人和俱在,基础、环境、条件并存。

形势逼人,机遇难得,不进则退,慢进也退。必须增强加快发展的危机感、紧迫感、责任感。市委、市政府立足实际,统筹全局,着眼长远,作出了实施名城复兴、百企强市、百园立农、百校兴教、城镇提质、城乡安居、收入倍增、生态建设、城乡清洁、平安创建等十大工程的决策部署。这是贯彻科学发展观和五位一体总布局的生动实践,是落实省委、省政府两件大事的重要举措,是实施转型发展、绿色崛起战略的主要抓手。全市上下要凝心聚力,团结拼搏,全力推进十大工程,走出一条全面协调可持续的路子,实现转型跨越可进位的目标。

三、2013年主要工作

今年是落实党的十八大精神的开局之年,是实施十大工程的起步之年,做好今年工作意义重大。政府工作的指导思想是:全面贯彻落实党的十八大精神,以转型综改试验区建设为统领,以项目推进年为抓手,以争先进位、负重赶超为总要求,围绕转型发展、绿色崛起发展战略,坚持统筹兼顾、协调发展、对标一流、稳中求进,大力推进工业新型化、农业现代化、市域城镇化、城乡生态化,保重点、保增长、保民生、保续建、保安全,全力抓好三件大事,打好四大硬仗,实施十大工程,推动经济社会持续健康发展,为实现“五市五同”目标、全面建成小康社会奠定坚实基础。

全市经济社会发展主要预期目标是:地区生产总值增长11%,规模以上工业增加值增长14.5%,全社会固定资产投资增长22%,财政总收入增长17%,公共财政预算收入增长17%,社会消费品零售总额增长15%,城镇居民人均可支配收入增长11%,农民人均纯收入增长14%,城镇新增就业岗位5.6万个,城镇登记失业率控制在4.2%以内,居民消费价格总水平涨幅控制在3.5%左右,城镇化率提高1.5个百分点。

约束性指标是:万元生产总值综合能耗下降3.4%,二氧化碳排放量下降3.7%,二氧化硫、化学需氧量、氨氮、氮氧化物排放量完成省下达任务,烟尘、粉尘排放量均下降2%,万元工业增加值用水量下降5.8%,人口自然增长率控制在6.5‰以内。

实现上述目标,必须全力实施十大工程。

(一)大力实施名城复兴工程,加快建设魅力城市。

古城承载着梦想,新城寄托着希望。要建好特色城,打好文化牌,提升大同知名度、美誉度和竞争力。

加强世界文化遗产和“四名”保护。坚持合理开发与永续利用相结合,真实性与整体性保护相结合,传统风貌保护与有机更新相结合,完善功能与增强活力相结合,加强云冈石窟和历史文化名城、名镇、名村、风景名胜区保护。继续推进云冈石窟五华洞窟檐保护及岩体加固和西城墙、代王府、四牌楼、魁星楼、传统民居保护工程,推进展览馆平移和联通公司迁建工程。做好浑源、左云等历史文化名城和天镇新平堡镇、大同县落阵营村等名镇名村保护工作。加强北岳恒山、大同火山群等风景名胜区保护、开发和利用。彰显大同厚重人文资源、丰富自然资源的优势和特色。

加快新区建设。进一步完善城市路网,建成27条43公里城市道路。按期建成太阳宫,基本建成博物馆、图书馆、美术馆、大剧院、体育中心。加快建设住宅和商业配套设施,完善功能,积聚人气。倾力打造集约、智能、低碳、绿色的一流新区,展示大同新形象。

发展文化旅游产业。用好世界文化遗产和历史文化名城金字招牌,文化唱戏,旅游拉动,激活古城,促进发展。建设文化科技产业基地和工业遗址创意产业园,完善晋华宫国家矿山公园。鼓励开发滑雪、温泉度假等冬季游乐项目。组织好第十二届中国(大同)云冈文化旅游节等一系列文化活动,不断扩大“中国古都·天下大同”影响力。

(二)大力实施百企强市工程,加快建设经济强市。

无工不富,无企不强。要坚持以煤为基、以煤兴业、以煤兴产、多元发展,以企业为主体、园区为载体、项目为实体、煤炭为本体,国有民营一起上,煤与非煤两手抓,二三产业同时干,抓大壮中,育小扶微,建立企业发展五个梯队方阵,激励企业奋力争先,跨越进位。市和每个县(区)重点支持发展100户企业。

以煤为基,多元发展。支持同煤集团冲刺世界500强,东周窑1000万吨矿井达产,金庄1000万吨矿井加快投产,实现大同地区1亿吨生产目标。加快中煤、晋能、山煤等企业矿井的复工建设步伐,建成标准化矿井16座,新增煤炭产量800万吨。浑源、矿区所属煤矿要完备手续,早日开工。加快推进同煤塔山2×60万千瓦、国电2×100万千瓦等火电项目建设,促进煤电联营、煤电一体。发展煤机制造、煤化工、煤建材、煤物流等产业。打造东小城等一批城市综合体,壮大华林等一批商贸名企,加快发展服务业。

加强园区经济。经济技术开发区要完善规划、理顺体制、扩区提质,发挥好国家级开发区体制机制优势。推进装备制造、医药化工、阳高龙泉等园区发展,积极创造条件申报省级园区。鼓励实力雄厚的企业采取托建托管模式,进行开发建设,把园区打造成要素集约、资源集聚、创新集成、产业集群的发展平台。

壮大县域经济。大同发展最大的潜力和空间在县域。各县(区)要立足资源禀赋、产业基础、人文环境,因县施策,有中生优,无中生有,明确发展定位,突出地域特色,整合资源要素,找准突破重点。城区、矿区要重点发展商贸、物流、餐饮等第三产业;新荣、浑源、灵丘要科学开发矿产资源加快发展;阳高、天镇、广灵等要重点发展特色农业和新能源产业;左云、南郊、大同县要重点服务好煤制气、甲醇、烯烃、多晶硅等大项目建设,快速提升经济总量。要研究好、争取好、叠加好、运用好综改试验、扩权强县、扶贫攻坚等政策,达到政策效应最大化,努力实现县域经济三年翻番。

发展民营经济。坚持非禁即入、非限即许,思想上放心放胆、工作上放手放开、政策上放宽放活,着力解决创业难、落地难、融资难问题。鼓励支持企业科技创新,增强核心竞争力。形成国有企业顶天立地、民营企业铺天盖地的发展局面。

(三)大力实施百园立农工程,加快建设农业大市。

发展农业园区,走园区引领带动的路子,是农村发展、农业增效、农民增收的必然途径。要以“一县一业”、“一村一品”为主攻方向,利用3年时间,规划建设七大类100个现代特色农业园,加快建设现代农业示范区。

加快园区规划建设。抓紧编制实施市县(区)园区建设规划和年度计划。完善园区基础设施,提升项目承载能力。打造阳高万亩标准园等设施农业、新荣森康等标准化养殖、南郊华晟等农产品加工、大同新发地等农产品物流、浑源春润等农业观光、天镇等高产创建、大同县黄花、广灵食用菌和灵丘核桃、左云小杂粮等特色种植园区。引导农业龙头企业、种养大户、农民专业合作社、家庭农场等新型农村经济组织参与园区建设,鼓励园区内农民在依法自愿前提下流转土地承包经营权,搞活经济、增加收入。与同煤、晋能、山煤等企业主动对接协调、落实产业扶贫项目,走出一条农业园区经济专业化、特色化、规模化的发展新路。

夯实园区发展基础。加强耕地保护,建设高标准基本农田8万亩,新增设施农业3万亩,改造中低产田20万亩、盐碱地5万亩。实施77.8万亩引黄雁同灌区节水改造工程。发展膜下滴灌2.94万亩。加快守口堡水库和天镇、阳高、广灵、南郊4个小型农田水利重点县(区)项目建设。抓好水库除险加固,确保安全稳定运行。

推进新农村建设。以硬化、绿化、亮化、美化为目标,进一步改善农村交通、卫生、居住环境。建成一批园企带动、异地搬迁、旧村整治、乡村旅游、中心村集聚等不同类型的美丽乡村和新型农村社区,推进新农村建设与城镇化良性互动。

(四)大力实施百校兴教工程,加快建设教育强市。

教育关系千家万户,关乎子孙后代。要以改革、规范、发展为主题,坚持硬件建设与软件提升并重,每年打造100所示范学校,办好布局合理、优质均衡、人民满意的教育。

深化办学体制和学校管理体制改革。推行联盟校、集团化办学,以优质学校带动薄弱学校,放大优质教育资源,促进教育均衡发展。实行中小学校长三年任期目标责任制,落实校长年度目标考核和后三位问责制度。逐步推行学校去行政化,实现校长职业化。建立教师交流制度,推进教师合理流动,努力实现师资均衡。激发学校活力,提高教育质量。

规范学校办学行为和教师从教行为。严禁义务教育阶段学校设置重点班、快慢班、特长班,切实减轻学生课业负担;严格小升初入学免试、就近、划片、分配制度,不得违反规定跨学区招生。实行初中入学全市同一天电脑统一编班制度。中考体育测试变人考为机考。开展“颂师德、正师风、强师资”主题活动。严肃查处教师乱补课、乱办班、乱收费行为。

加快学校建设和教育体系建设。完成大同一中初中部等7所学校续建工程,推进特教学校等10所学校工程建设,完成县(区)58所学校的改扩建工程。提升中小学信息资源应用水平,实现示范校“校校通、班班通、人人通”。加强学校运动场地建设,确保学生每天锻炼一小时。推进校园监控系统全覆盖,创建平安校园。办好县办高中,吸附学生本地上学,遏制生源流失现象。大力发展职业教育。鼓励和规范发展民办教育。支持大同大学发展。

(五)大力实施城镇提质工程,加快建设宜居城市。

城镇化是经济持续快速增长的巨大引擎和最大潜力。要坚持规划引领,建设管理并重,扩容提质并举,加快构建以中心城市为核心、大县城为重点、重点镇为基础的城镇体系,提升市域城镇化质量。

强化城乡规划指导。加快编制城镇体系规划、都市区规划、城市总体规划、详细规划、保护规划和专项规划等各类规划,抓紧评审报批,健全规划体系,确立法定地位,发挥指导作用。严格规划审批,加强规划执法,确保规划实施。创新体制机制,加强统筹协调,实现城乡规划、国民经济社会发展规划、土地利用规划、环境保护规划、园区(产业)发展规划五规合一。

加强城市建设管理。坚持依法征收、阳光征收、和谐征收,按照政策不变、主体不变、任务不变、力度不变的要求,加快推进土地房屋征收征用,为城市建设提供保障。今年完成征收1.2万户。积极探索以城养城、以城建城新路径,整合城市资源,盘活城市资产,变资源为资产,变资产为资本,多渠道筹措资金,破解城市建设资金瓶颈。加快数字城管信息平台建设,积极申报国家智慧城市(区)试点。不断提高城市精细化、智能化、科学化管理水平。

实施大县城战略。以城中村改造、保障性住房建设为切入点,搞好县城旧区综合整治,加快建设路、水、气、热等市政基础设施,办好中学、医院、“三馆一场”等公共事业,推进“四改四变三集中”,做大做强县城,提升县城综合承载能力,增强县城产业发展、公共服务、吸纳就业、人口集聚功能。促进人口向县城集中、基础设施和公共服务设施向农村延伸。打造统筹城乡发展桥头堡,实现就地低成本城镇化。

加强重点镇建设。以南郊云冈镇等6个省级重点镇、大同县周士庄镇等9个市级重点镇为重点,编制完成近期建设规划,推进“五建设两整治”,培育一批集群发展型、新区建设型、旧区提质型、园区拉动型、乡村旅游型等不同特色小城镇。

(六)大力实施城乡安居工程,加快建设康居大同。

安居才能乐业。要大力开展住房保障管理年活动,进一步提高保障性住房建设管理水平,确保建设好、分配好、管理好、居住好。

加快城镇保障性住房建设。开工建设1.99万套,续建8.06万套,竣工5.23万套。严格落实保障性住房管理和廉政风险防控6+1制度。继续实行土地计划单列,做到应保尽保。加快工程进度,确保如期交工。建成项目要及时验收,确保质量合格、设施配套、功能齐全。坚持公开公平公正分配,及时回迁安置被征收群众。加强物业管理,提高服务水平。

抓好农村安居工程建设。继续推进农村困难家庭危房改造,围绕对象认定、质量监督、资金监管等环节,完善工作机制,确保完成任务。开展农村住房抗震改建试点,以县为责任主体、乡镇为实施主体,规划先行、农民自愿、统一组织、成片推进,指导农户通过新建或加固的方式,建设安全、舒适、节能、美观的住宅。完成左云、阳高等5县4000户改建任务。改建试点补助资金由省市县分担,国家贫困县由省市按7:3比例每户补助3万元。

(七)大力实施收入倍增工程,加快建设幸福大同。

增加收入是人民群众最现实的利益,更是政府工作义不容辞的职责。要千方百计,多措并举,全面实现收入倍增目标。

扩大就业规模。实施就业优先战略和更加积极的就业创业政策。拓宽城镇就业渠道,做好城镇新增就业、扶持创业和农村劳动力转移工作。鼓励民营企业提供就业岗位。建设市、县、乡、村四级公共就业服务体系和就业信息服务网络。扶持3000人实现创业,组织城镇登记失业人员技能培训2万人,组织农村劳动力转移就业技能培训2万人,开展创业培训3500人,培养技能人才1.2万人。转移农村劳动力3万人。

提高城乡居民收入。建立职工工资标准正常增长机制,推行企业工资集体协商制度,加强各类企业工资分配监管,严格执行最低工资标准。依法保障农民对承包土地占有、使用、收益等权利,鼓励农民创业、置业、投资、理财,增加财产性和转移性收入。努力提高农民工资性收入,确保农民人均纯收入增幅高于城镇居民收入增幅。

完善社会保障体系。扩大城镇职工基本养老保险覆盖面,继续调整提高企业离退休人员养老保险待遇,完善新型农村社会养老保险和城镇居民养老保险、城乡最低生活保障及社会救助制度,加快发展社会福利和慈善事业。推进社会保障信息网络建设,发放社会保障卡150万张,基本实现人人持卡。深化医疗卫生体制改革,提高新型农村合作医疗和城镇居民医疗保障水平。完善失业保险制度。扩大大病救助范围,提高救助标准,防止因病致贫、因病返贫。发挥社会保障在改善民生、发展经济、促进稳定中的稳压器作用。

(八)大力实施生态建设工程,加快建设绿色大同。

生态的差距是最大的差距。要大力开展“三城联创”,努力实现生产空间集约高效、生活空间宜居适度、生态空间山清水秀。

推进节能降耗。以创建国家新能源示范城市为契机,加强工业、建筑、交通三大领域节能工作。抓好58户省定重点节能企业能耗监测监控。强化淘汰落后产能督查调度,抓好高耗能落后设备的按期拆除。完成既有建筑节能改造30万平方米,新建建筑节能标准执行率达到100%。加快推进供热分户计量、按量收费工作。优先发展公共交通,更新公交车150辆,巩固出租车油改气成果。充分发挥公共机构节能示范作用。推广应用节能、节水、节材技术和产品。积极推进风、光、地热等清洁能源开发利用。

推进污染减排。以创建国家环保模范城市为契机,强化污染减排综合性措施。加大实施脱硝、脱硫、烟尘和粉尘治理力度,实施PM2.5空气质量评价体系,确保空气质量达到国家标准。实施断面水质生态考核补偿机制,完成御河、十里河、口泉河、南洋河等河流治理。完成御东污水处理厂和东郊、西郊污水处理厂新改扩建工程。新增集中供热面积800万平方米。推进和探索排污权、碳排放权交易。

推进造林绿化。以创建国家园林城市为契机,全面提升城市绿化水平。完成御东新区公共活动走廊、御河两岸景观、梓家村森林公园、儿童公园、城墙周边等园林绿化建设。新增建成区绿化面积120万平方米,提高绿化覆盖率1个百分点。加大县城绿化力度,力争实现省级园林县城“零突破”。认真实施国家重点生态建设工程和省造林绿化六大工程,完成造林32.36万亩,提高森林覆盖率1个百分点。突出通道绿化、村庄绿化、庭院绿化,加强身边增绿,使有人的地方先绿起来,让人民群众最大限度享受生态建设成果。

(九)大力实施城乡清洁工程,加快建设美丽大同。

城乡清洁工程是环境工程、民心工程、作风工程。一个城市既要外表靓丽、获得游客好评,更要内里干净、赢得居民认可,让广大居民实实在在地看到变化、分享成果。城乡清洁既要打赢歼灭战,更要打好持久战,实现城市环卫管理精细化、农村环卫保洁常态化、城乡垃圾处理一体化、环境卫生服务均等化。

加强“五治五进”城市环境综合整治。规划设置停车场、停车带,缓解停车难;建设集贸市场,规范摊点摆设;落实“门前五包”,加强垃圾收运;规范广告设置,清除视觉污染;加强工地管理,做到文明施工。整治背街小巷、老旧社区、城中村环境卫生,实现环卫保洁全覆盖、市容市貌上水平。开展“最美环卫工人”评选活动。不断提高环卫工人待遇。

推进“四堆五净”乡村清洁。按照“村收集、乡转运、县处理”的垃圾收运处理模式,统一配备设施,统一调配车辆,统一选聘人员,统一考核评比,全面改善乡村环境。重点打造景区景点和县城周边村、乡镇驻地村、沿河沿路村,展现农村新面貌。完成浑源、灵丘、阳高垃圾处理场建设,实现“县县有”目标。

城乡清洁工程是改变生活习惯、改善生活环境的一场革命,需要全市人民共同参与和不懈努力。人人都要从自己做起、从小事做起、从清洁做起,养成良好习惯,共建美好家园。

(十)大力实施平安创建工程,加快建设平安大同。

安全是生产的前提,稳定是发展的基础。要始终坚持安全第一,不断强化“一岗双责”,严格落实两个主体责任。

持之以恒抓好安全生产。深入开展安全生产专项整治,不断创新安全生产监管方式和手段,建立安全监管有奖举报制度。切实加强煤矿、非煤矿山、危险化学品、道路交通、建筑施工等重点行业、重点领域和重点县(区)、重要节点的隐患排查整治。加强食品药品安全监管工作,加大消防安全事故查处力度。严格执行“四不放过”原则,落实安全生产“一票否决”制度。强化安全培训,夯实基层基础,推动安全生产形势由稳定好转向根本好转转变。

全面加强和创新社会管理。推进“天眼工程”建设,完善社会治安防控体系。抓好信访和矛盾调处工作,畅通群众诉求渠道,依法规范信访秩序。创新流动人口和特殊人群服务管理,建立“三级平台”、“四级网络”基层社会服务管理体系,实行社区网格化管理。

严厉打击非法违法行为。始终保持高压态势,严厉打击私挖滥采、非法生产和违法建设,严厉打击车辆超限超载和公路“三乱”,严厉打击各种违法犯罪活动。

在全面推进十大工程的同时,要统筹做好国防动员、人民防空、双拥优抚、人口计生、妇女儿童、老龄、残疾人、红十字、民族宗教、外事侨务、档案史志、防震减灾、应急管理、气象、统计、体育等各项工作。

各位代表,为民造福是政府工作的最终目标,是检验政府工作的根本标准。今年,市政府要为民办好十件实事:

新建城市棚户区改造安置房8550套;

改造农村困难家庭危房2.08万户;

提高农村7.5万人饮水安全标准;

完成1166个行政村街道亮化;

建设13个社区公园,绿化19个安置区;

完成异地搬迁农村特困人口4140人;

新建30所标准化幼儿园,改扩建42所村级幼儿园;

为所有环卫工人每天补助早餐费6元;

为所有行政村统一配备清扫保洁人员;

为城市低保对象每月提标30元,为农村低保对象每月提标24元。

我们还要举全市之力,办好8月份举办的国际太阳能十项全能竞赛。要高度重视,周密安排,精心组织,切实抓好场馆建设、环境整治、赛事组织、接待服务、安全保卫等工作,确保大赛圆满成功,并以此为契机,推动我市新能源产业发展。

四、落实与保障

十大工程涵盖了全市经济社会发展的主要方面,是一项事关全局的系统工程。必须细化措施,优化要素,强化落实,全力推进。

(一)以转型综改试验区建设为统领落实十大工程。

转型综改试验既是关系全省大局的首要任务,也是我市推进十大工程的统领和保障。我们要紧紧抓住这一难得发展机遇,以开放包容、崇实尚新的大同精神和敢为人先、先行善试的英雄胆识,逢山开路、遇河架桥,鼓励创新、宽容失败,以更大的决心和更多的办法推进转型综改试验,发挥改革最大红利作用,化解矛盾,克服困难,推进十大工程顺利实施。

省委、省政府已批准下发转型综改试验实施方案和2013年行动计划,标志着转型综改试验区建设的全面铺开、纵深推进。要把我市列入全省盘子中的重大改革、重大事项、重大项目,作为全市转型综改试验和推进十大工程的重中之重,创新“五规合一”规划统筹协调机制,深化行政审批制度改革,推进同煤、中海油低变质烟煤清洁利用气电热一体化项目,同煤60万吨甲醇、60万吨烯烃项目,大同协和新能源多晶硅及光伏产业循环经济项目。

尽快制定市县(区)两级转型综改试验实施方案和年度行动计划。灵丘县作为省级试点要创新土地管理和金融发展机制,新荣区作为市级试点要完善促进循环经济发展、生态环境治理投入机制。各县(区)都要按照转型综改试验的要求,设定目标值,绘好路线图,明确时间表,大胆改革、锐意进取,全面完成转型综改试验任务,充分发挥对全市经济社会发展,特别是十大工程的统领作用,努力走出一条资源型老工业城市转型跨越的路子。

(二)以项目推进年为抓手落实十大工程。

项目是十大工程的载体。要按照“六位一体、统筹推进”的要求,扎实开展项目推进年活动,充分发挥重点工程在保增长、调结构、惠民生中的火车头、主力军、排头兵作用。

建立健全并严格落实市县(区)两级四大班子领导联系重点企业重点项目制度、市直部门对口支持重点企业制度、“三个24小时”直通车制度、政府领导按月调度推进制度、对县(区)和市直部门进行考核排名通报等制度。加快推进重大项目建设,特别是要抓好同煤塔山循环经济园等十大在建标杆项目和大张客运专线等十大前期标杆项目。以大项目促进大发展,多项目促进快发展,好项目引领转型发展。

加大招商引资引才力度,以企招商,以商招商,营造招商亲商、安商富商的浓厚氛围,引进更多的世界500强和中国500强在同投资,吸引更多的人才在同创业发展。培育和扶持本地企业发展。创造良好环境,实现资金回流、老板回家、情感回归、人才回乡。探索发展“飞地经济”,加快产业承接步伐。争取金融机构扩大信贷规模,鼓励企业利用资本市场融资,合理引导利用民间资本,多渠道筹措建设资金。

(三)以项目化管理为手段落实十大工程。

实施十大工程,要按照“五个一”和“十个有”要求,实行项目化管理。制定市、县(区)、乡(镇)三级“一事一表”,建立横向到边、纵向到底、责任到人的推进机制。

对标一流定目标。项目化管理的前提是目标正确。法乎其上,得乎其中。要瞄准一流,以终为始,善于创新,敢于超越,确保十大工程管理一流、质量一流、效益一流。

一事一表做计划。项目化管理的精髓在于精细。天下大事,必作于细。要通过一事一表,理清思路,分解任务,明确路径,落实责任,消除工作盲区,不留责任空白,实现精细管理。

按月调度抓落实。项目化管理的根本在于落实。因行而至,经为而成。要按照业务服从原则,实行分管领导一月一调度、一月一推进、一月一排队、一月一通报制度,掌握进度,解决问题,推进工作。

活力曲线抓考核。项目化管理的关键在于考核。勋赏千金,无功不与。要加大对各县(区)、各部门“三合一”考核力度,定期排队通报,激励先进,鞭策中游,淘汰落后。充分发挥考核的指挥棒、风向标、助力器作用,激励全市上下在十大工程主战场见水平、比高低、论英雄。

(四)以政府自身建设为保障落实十大工程。

政府是十大工程的组织者和推动者,要加快转变职能,提高行政效能,努力建设人民满意的政府。

敬畏人民,建设服务政府。始终牢记宗旨,以人民群众的呼声为第一信号,以人民群众的需求为第一选择,以人民群众的满意程度为第一标准。坚持群众路线,问政于民、问计于民、问需于民,凡是涉及人民群众利益的决策都要充分听取意见,凡是损害人民群众利益的做法都要坚决防止和纠正。建立行政投诉快速反应机制,不断优化发展环境。

敬畏法律,建设法治政府。自觉接受人大法律监督和政协民主监督,虚心接受舆论监督和群众监督。坚持依法行政,严格遵循职权法授、程序法定、行为法限、责任法究的原则,不断加强制度建设,严格按照法定权限和程序行使权力,提高运用法治思维和法治方式履行职责的能力。理顺行政执法体制,规范行政执法行为,强化行政执法监督。

敬畏权力,建设责任政府。权力代表责任,有权必有责任。要勇于负责,敢于担当,直面矛盾,解决问题。面对人民,俯首甘为孺子牛;面对责任,不待扬鞭自奋蹄。以牛的力度,马的速度,做到又好又快。

敬畏纪律,建设廉洁政府。严格落实中央八项规定,改进作风,勤政为民,既要把权力关进制度的笼子里,用制度管人、管权、管钱、管事;又要把权力晒在监督的阳光下,实行政务公开。严格行政问责。强化党风廉政“一岗双责”,加强廉政教育,建设廉政文化。不断加大审计监督力度。全面改进公务活动安排,严控“三公”经费,厉行勤俭节约,反对铺张浪费。

各位代表,我们的任务艰巨而繁重,使命崇高而神圣,前景光明而美好。让我们紧密团结在以习近平同志为总书记的党中央周围,在省委、省政府和市委的正确领导下,团结带领全市人民,以更加强烈的责任意识,更加饱满的精神状态,更加务实的工作作风,推进伟大事业,创造崭新业绩,开创美好明天,为实现“大同梦”而努力奋斗!
